# Tai Chi for patients with mild cognitive impairment: A protocol for a systematic review and meta-analysis of randomized controlled trials

**DOI:** 10.1097/MD.0000000000017118

**Published:** 2019-10-04

**Authors:** Juan Yang, Tony Y. Chon, Guangxi Li, Molly J. Mallory, Sara E. Bublitz, Alexander Do, Lizu Xiao, Donglin Xiong, Brent A. Bauer

**Affiliations:** a Division of General Internal Medicine, Mayo Clinic, Rochester, MN; b Department of Pain Medicine, Shenzhen Nanshan People's Hospital, Guangdong Medical University, Shenzhen; c Guang’anmen Hospital, China Academy of Chinese Medical Sciences, Beijing, China; d Division of Pulmonary and Critical Care Medicine, Mayo Epidemiology and Translational Research in Intensive Care, Rochester, MN.

**Keywords:** mild cognitive impairment, protocol, randomized controlled trial, review, Tai Chi

## Abstract

**Background:**

Mild cognitive impairment (MCI) is an intermediate stage between the cognitive changes of normal aging and early dementia. Tai Chi (TC) may be particularly beneficial to patients with MCI due to its whole-body coordination characteristics. This systematic review protocol aims to outline the methods that will be used to assess the comparative effectiveness and safety of TC for MCI through a systematic review and meta-analysis.

**Methods:**

A systematic review will identify and evaluate randomized controlled trials (RCTs) that examined the effects and safety of TC compared to a placebo, conventional treatment, and no treatment on cognitive function in individuals with MCI. Studies from databases of MEDLINE, PubMed, Embase, Global Health, Cochrane Library, and Scopus from January 1990 to March 2019 reported in English will be searched. Two independent reviewers will screen the studies for inclusion with the eligibility criteria and extract data. Risk of bias of individual studies will be assessed in line with Cochrane risk of bias tool. The overall quality of cumulative evidence will be assessed using selected Grading of Recommendations, Assessment, Development, and Evaluations criteria. Statistics will be used for heterogeneity assessment, sensitivity analysis, data synthesis, generating funnel plots, and subgroup analysis. Meta-analysis will be performed, if sufficiently homogeneous studies are found. A narrative synthesis will be conducted, grouping studies by exposure and outcome definitions, and describing any differences by subgroups.

**Results:**

This study will provide practical and targeted evidence in investigating the impact of TC exercise for individuals with MCI.

**Conclusion:**

The findings of our study will provide updated evidence to determine whether TC is an effective intervention to patients with MCI.

**Trial registration number:**

International Prospective Register for Systematic Reviews (PROSPERO) number CRD42019125104.

## 1 Introduction

Mild cognitive impairment (MCI) is defined as a stage with subjective and objective decline in cognition function which does not meet the criteria for a diagnosis of dementia.^[1]^ It refers to a borderland between normal age-related cognitive changes and dementia.^[2]^

Literature has indicated that MCI incidence was 6.7% for elderly ages 60 to 64, 8.4% for 65 to 69, 10.1% for 70 to 74, 14.8% for 75 to 79, and 25.2% for 80 to 84.^[3]^ It is not only the 1st cognitive expression of dementia, but it is also associated with other disease processes such as systemic, psychiatric, neurologic, or neurodegenerative disorders.^[4]^ The presence of MCI is independently associated with an increased incidence of critical illness hospital admissions, which threatens the quality of life among the elderly.^[5]^ Nowadays, approximately 22.2% of people in the United States aged 71 years or older suffer from MCI.^[6]^ In the world, nearly 47 million people are suffering from dementia, and every year about 10 million new cases are diagnosed. This number is projected to nearly triple to more than 130 million by the year 2050.^[7]^

Strategies to delay the progression of MCI are being sought to meet this challenge. Currently, pharmacologic treatments to improve cognition or slow the disease progression have had limited effectiveness.^[8]^ Physical activity is a well-researched behavioral intervention for cognitive function, which can reduce the relative risk of dementia by 28%.^[9]^ Hypothesized mechanisms include direct effects on the brain, such as increasing vasculature and production of neurotrophic factors, which might benefit neuronal repair, growth, and plasticity.^[10]^ Previous reviews have reported that the progression of cognitive decline could potentially be prevented or slowed with a physically active lifestyle.^[11]^ A 2011 meta-analyses of prospective studies indicated that physical exercise may facilitate neurotrophic factors production, attenuate neurodegenerative processes progression, and reduce vascular risk factors resulting in cognitive improvement.^[12]^ Studies have shown that exercise training for 6 months is likely to enhance cognitive measures, while the optimal type of physical activity/exercise remains unclear.^[3,13,14]^ Tai Chi (TC) or Tai Ji, which origins predate the 17th century, combined both Chinese martial art and health regimens into a common set of core principles, movements, and exercises.^[15]^ It may be particularly beneficial for older adults with MCI, given the whole-body coordination of continuous rhythmic movements along with cognitive activities such as movement memorization.^[16,17]^

Over the last 10 years, TC has gained popularity as a therapeutic physical activity for elders with MCI,^[18]^ cognitive impairment,^[19]^ and dementia.^[20]^ A number of nonrandomized and randomized clinical trials have shown the therapeutic effects of TC on cognition. Some reviews have explored the generalized correlation between TC and cognitive function^[21–23]^ with 3 evaluating global cognitive function,^[22,23]^ and 1 narrative review to cognitive function.^[21]^ No review has comprehensively and quantitatively synthesized the literature analyzing the direct correlation with TC and improved mild impaired cognitive function. Those previous reviews and methodologies are overviewed in Table 1. We propose a systematic review to determine the effectiveness and safety of TC in treating MCI among older adults.

**Table 1:**
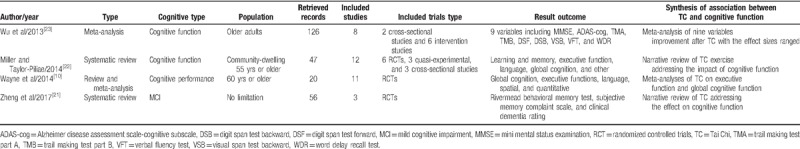
Overview of previous literature reviews investigating the impact of Tai Chi on cognitive function.

## 2 Methods

### 2.1 Study registration

This systematic review protocol was registered on the PROSPERO International Prospective Register of Systematic Reviews with on February 12, 2019 (registration number: CRD42019125104). Available at: https://www.crd.york.ac.uk/prospero/#recordDetails. In addition, our review will comply with the guidelines of the Preferred Reporting Items for Systematic Reviews and Meta-Analyses (PRISMA) statement guidelines.^[24]^

### 2.2 Inclusion criteria for study selection

#### 2.2.1 Inclusion/exclusion of articles

Eligible articles must meet the following criteria to be included in the review: reported research investigates human participants; original research publication in a peer-reviewed journal; randomized controlled trials (RCTs) published from January 1990 to March 2019; subjects with MCI according to the adaptations of criteria suggested by Petersen RC^3^; participants in control group had no exercise or low intensity exercise such as muscle toning or stretching; the effect of TC exercise over cognitive function was the primary or secondary outcome; available in English; non-RCTs including cohort, case series report, and case–control will be excluded.

#### 2.2.2 Type of participants

Subjects aged 55 years or older, diagnosed with MCI will be included in the review. There will be no restriction on sex, ethnicity, or disease duration.

#### 2.2.3 Type of interventions

Trials assessing the impact of TC on MCI will be included. Any type of TC intervention will be included, regardless of duration and treatment frequency.

#### 2.2.4 Type of comparators

Studies for the effect and safety of TC on MCI will be included if they compare TC to any control group such as placebo, conventional treatment, and no treatment. TC adjunctive therapy in combination with conventional treatment will be compared with conventional treatment alone. This review will exclude pharmacologic agents as comparator.

#### 2.2.5 Outcome measurements

The primary aim is to review the literature and synthesize relevant data to investigate the association between TC and MCI in adults comprehensively. Test variables such as general cognitive function, executive function, and memory function such as the MMSE, ADAS-cog, or WAIS will be included. If possible, a secondary examination of adverse events of TC will also be assessed.

### 2.3 Search strategy

The search strategy was designed and conducted by the study's principal investigator and coinvestigators in collaboration with an experienced librarian. Controlled vocabulary supplemented with keywords was used to search for studies involving impact of TC exercise on cognitive decline in older people. A comprehensive search of several databases MEDLINE, PubMed, Embase, Global Health, Cochrane Library, and Scopus from January 1990 to March 2019, English language only will be sought. Search terms were used: (Tai Chi OR Taichi OR Tai-ji, Taijiquan OR Taiji OR Tai Ji OR Tai Chi OR Tai Chi Chuan) AND (Mild Cognitive OR Mild Cognitive Impairment∗, Cognitive Impairment∗, Mild OR Impairment∗). Retrieved Records from the electronic databases will be downloaded to the software EndNote X8 for screening independently by 2 researchers. Two independent reviewers will screen the abstracts of all the articles against the eligibility criteria. The review protocol will be designed and conducted according to the Cochrane Handbook for Systematic Reviews of Interventions and reported according to the Preferred Reporting Items for Systematic reviews and Meta-Analyses Protocols (PRISMA-P) statement guidelines.^[25]^

### 2.4 Data collection and analysis

#### 2.4.1 Selection of studies

**Figure 1:**
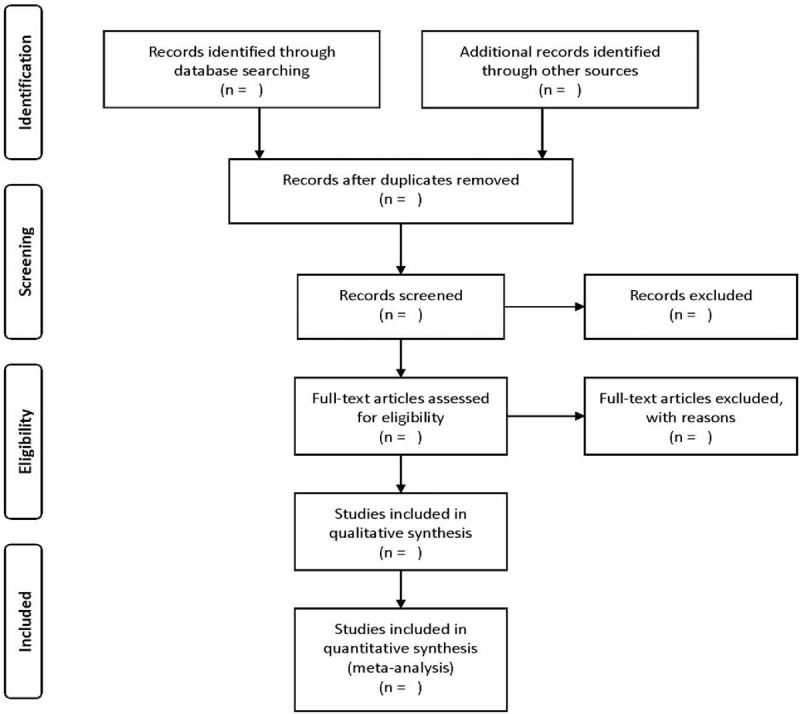
The Preferred Reporting Items for Systematic Review and Meta-Analysis flow diagram.

Identified studies will be uploaded to the software reference manager EndNote X8 for screening. Two reviewers will independently screen the abstracts of all the articles against the eligibility criteria. If there is any discrepancy on an article's inclusion, a 3rd reviewer will be consulted independently. Retained articles will be subject to full text review. A PRISMA flow diagram^[25]^ will be used to document the whole trial selection process (Fig. 1).

#### 2.4.2 Data extraction

Data extraction will be performed independently by 2 reviewers using Excel spreadsheet software. Extracted data will include important characteristics of the studies including genera information, methods, participants, and interventions. Any discrepancy and uncertainty will be resolved by consensus between the 2 reviewers or by asking the third author to make a final decision.

#### 2.4.3 Assessment of bias in the included studies

Each trial included in the review will be independently assessed by 2 reviewers for potential biases. For any disagreement, a 3rd reviewer will help to confirm the final assessment. Original article authors will be contacted (when possible), if there are insufficient details to confidently assess the risk of bias. Based on the Cochrane risk of bias tool version 5.1.0,^[26]^ each article will be rated as “high,” “unclear,” or “low” for performance, attrition, and reporting bias on the following domains: random sequence generation; allocation concealment; blinding of participants and personnel; blinding of outcome assessment; incomplete outcome data; selective reporting; and other bias. Trials will be classified according to the following categories: high risk: studies with one or more domains at high risk of bias; low risk: studies with all domains at low risk of bias. A risk of bias summary table will be produced.

#### 2.4.4 Quality assessment

The certainty of the overall evidence and recommendation strength will be assessed to each trial with the Grading of Recommendations Assessment, Development, and Evaluation (GRADE) approach.^[27]^ The evaluation domains include 5 criteria: risk of bias, inconsistency, indirectness, imprecision, and publication bias. Each certainty of evidence will be graded as “high” (further research is very unlikely to change the confidence in the effect estimates), “moderate” (further research is likely to have an important impact on the confidence in the effect and may change the estimate), “low” (further research is very likely to have an important impact on the confidence in the effect and is likely to change the estimate), or “very low” (any estimate of the effect is very uncertain). The assessment will be reported in GRADE summary of findings tables in the end. Two researchers will rate risk of bias of each trial independently. Any discrepancies will be resolved by discussion and consensus.

#### 2.4.5 Measures of treatment effect

The continuous data will be expressed as mean difference (MD) or standard MD (SMD) with 95% confidence intervals (CIs) and the dichotomous outcomes will be estimated by the risk ratio with 95% CIs.

#### 2.4.6 Assessment of heterogeneity

Heterogeneity will be assessed by visual inspection of the forest plots and detected by standard Chi-squared test and *I*^2^ statistic. Sensitivity analysis and subgroup analysis will be selected to detect the possible reason of substantial heterogeneity.

#### 2.4.7 Assessment of reporting bias

Heterogeneity sources will be explored by meta-regression method to compare summary estimates from different study levels, such as population age, MCI diagnosis, and study design. The most biased trials with 2 domains or over are will be excluded in sensitivity analysis for high risk of bias. If study numbers are sufficient, the risk of publication bias will be investigated with a funnel plot.

#### 2.4.8 Data statistics

All collected information and data will be organized and stored in Excel databases. All RCTs data will be entered into RevMan5.3 to synthesize the evidence. Study characteristics will be summarized: publication details (authors, publication year, and location), sample size, characteristics, participants’ inclusion and exclusion criteria, mean (SD) or median (interquartile range) for continuous variables and frequencies (%) for categorical variables. Any discrepancy that cannot be resolved from the data provided will result in a request from the original article author for additional data. Meta-analysis will be performed if sufficient data are available and the studies/methods are sufficiently homogeneous regarding the interventions and outcomes. Statistical tests of heterogeneity (Chi-squared and *I*^2^) will be applied. Effect estimates will be weighted by their variance inverse, regarding greater weight to larger trials. If a meta-analysis is not conducted for any outcome due to insufficient data, a qualitative synthesis of the findings from the included studies will be provided, structured around the intervention type, population characteristics, outcome result, and intervention content. Random-effects models will be used to calculate effect sizes and 95% CIs. If sufficient data are available, additional subgroup analyses with similar research questions will be carried out for studies, which will investigate differences between TC type (e.g., Yang-style simple form of TC, Sun style TC, 24-style TC, Taoist TC), severity of cognitive impairment (e.g., MCI, moderate to severe impairment, and dementia), study design (e.g., control group such as placebo, conventional treatment, and no treatment) and quality and risk of bias. If data are unavailable, a narrative description of these studies will be provided.

## 3 Conclusion

This review protocol is described for a systematic review and meta-analysis to determine a best estimate of the association between TC and MCI in elderly. It clearly states the types of trials, participants, interventions and outcomes to be included, as well as the search strategy, data sources, extraction methods, and data analysis. One of the strengths of the proposed study is the proposed review will be the 1st systematic analysis of studies that have examined the effects of TC on cognitive function among older adults. Our search strategy will include published and unpublished studies. Our proposed systematic review and meta-analysis will synthesize the available evidence using rigorous methods and provide evidence in support or against the hypothesis that TC is effective and safe to treat MCI among older adults, which will shed light on the effects of TC on cognitive function and permit identification of evidence gaps, therefore informing clinical decision making and guide future research initiatives.

## Acknowledgment

The authors thank the experienced librarian, Leslie C. Hassett, Mayo Clinic Rochester Campus for the literature research and the reviewers of the manuscript for their constructive feedback.

## Author contributions

**Conceptualization:** Juan Yang, Brent A. Bauer.

**Data curation:** Juan Yang, Lizu Xiao.

**Formal analysis:** Juan Yang, Donglin Xiong.

**Funding acquisition:** Donglin Xiong, Brent A. Bauer.

**Investigation:** Juan Yang, Brent A. Bauer.

**Methodology:** Juan Yang, Lizu Xiao, Donglin Xiong.

**Project administration:** Brent A. Bauer.

**Resources:** Brent A. Bauer.

**Software:** Juan Yang, Lizu Xiao, Donglin Xiong.

**Supervision:** Brent A. Bauer.

**Visualization:** Juan Yang.

**Writing – original draft:** Juan Yang.

**Writing – review & editing:** Tony Y. Chon, Guangxi Li, Molly J. Mallory, Sara E. Bublitz, Alexander Do, Brent A. Bauer.
